# Resistive switching of Au/ZnO/Au resistive memory: an *in situ* observation of conductive bridge formation

**DOI:** 10.1186/1556-276X-7-559

**Published:** 2012-10-08

**Authors:** Chung-Nan Peng, Chun-Wen Wang, Tsung-Cheng Chan, Wen-Yuan Chang, Yi-Chung Wang, Hung-Wei Tsai, Wen-Wei Wu, Lih-Juann Chen, Yu-Lun Chueh

**Affiliations:** 1 Department of Materials Science & Engineering, National Tsing Hua University, No. 101, Sec. 2, Kuang-Fu Rd., Hsinchu, 30013, Taiwan; 2 Department of Materials Science & Engineering, National Chiao-Tung University, No. 1001, University Rd., Hsinchu, 30013, Taiwan; 3 Center For Nanotechnology, Material Science, and Microsystem, National Tsing Hua University, No. 101, Sec. 2, Kuang-Fu Rd., Hsinchu, 30013, Taiwan

**Keywords:** Real-time observation, Au/ZnO/Au, Conducting bridge, Au nanoparticles

## Abstract

A special chip for direct and real-time observation of resistive changes, including set and reset processes based on Au/ZnO/Au system inside a transmission electron microscope (TEM), was designed. A clear conducting bridge associated with the migration of Au nanoparticles (NPs) inside a defective ZnO film from anode to cathode could be clearly observed by taking a series of TEM images, enabling a dynamic observation of switching behaviors. A discontinuous region (broken region) nearby the cathode after reset process was observed, which limits the flow of current, thus a high resistance state, while it will be reconnected to switch the device from high to low resistance states through the migration of Au NPs after set process. Interestingly, the formed morphology of the conducting bridge, which is different from the typical formation of a conducting bridge, was observed. The difference can be attributed to the different diffusivities of cations transported inside the dielectric layer, thereby significantly influencing the morphology of the conducting path. The current TEM technique is quite unique and informative, which can be used to elucidate the dynamic processes in other devices in the future.

## Background

Resistive random access memory (ReRAM) is one of the most significantly nonvolatile memories because of its fast switching speed, low power consumption, excellent endurance, and easy integration with current device processes [1,2]. Among the materials for ReRAM application, metal oxides have attracted an increasing interest in material choice because of controllable compositions. Therefore, making a high-quality metal oxide layer with a bistable resistance state is a key issue in achieving a high-performance ReRAM device. A typical configuration of the ReRAM device usually consists of metal/insulator/metal structures, and the operation is based on the switching of high resistance state (HRS) and low resistance state (LRS) (off and on states) after a larger bias was applied due to the formation of a conductive bridge/path [3]. In general, the switching behaviors can be classified into two types in terms of current–voltage (*IV*) behaviors, namely bipolar and unipolar switching [4,5]. The bipolar resistive switching shows a directional resistive switching depending on the polarity of the applied voltage, while the unipolar resistive switching depends on the amplitude of the applied voltage without any polarity.

Zinc oxide (ZnO) has many superb characteristics in optics and electronics, such as a direct bandgap of approximately 3.37 eV, adjustable electrical properties by doping with different dopants, and low synthesis temperature [6,7]. ZnO-based thin film ReRAM devices were found to have promising resistive switching characteristics with either unipolar or bipolar resistive switching behaviors, while the detailed switching behavior is still not clear. Therefore, unveiling the switching behavior of ReRAM based on ZnO system is imperative to shed light on the fundamental understanding of device operation, enabling the further exploration of ReRAM devices.

Recently, the existence of conductive bridges/paths has been confirmed and observed by different methods. Pan et al. used transmission electron microscopy (TEM) to observe the formation of the conductive bridge/path after a couple of set/rest processes [8-12]. Takimoto et al. used conducting atomic force microscopy to locate the formation of the conducting filament [13,14]. However, these methods for the observation of the conducting filament formation can only be considered as *ex situ* observations. Some controversies regarding resistive switching mechanisms remain, and the underlying physical mechanism of the resistive switching is still not fully understood.

Consequently, a direct observation of conductive bridge/path formation in real time is imperative. In this regard, we demonstrate a *real-time* observation of a conductive bridge/path formation in the Au/ZnO/Au system inside the TEM to clarify how the conducting bridge/path formed between two electrodes, including set and reset processes when the resistance is changed from high resistance to low resistance states in the Au/ZnO/Au system. To realize this goal, it is important to directly observe *in situ* images in real time [15-17]. The switching mechanisms of the Au/ZnO/Au device in ultrahigh vacuum and air conditions were investigated for comparison.

## Methods

### Fabrication of TEM chip for *in situ* observation

Si substrates were cleaned by standard processes. Si_3_N_4_/SiO_2_ thin film layers with thicknesses of 80/20 nm were deposited on both sides of a Si substrate with a low-pressure chemical vapor deposition (LPCVD) system at 780 °C in a vacuum of 350 mTorr. Position of the membrane area was defined by photolithography on the back side of the chips, and the Si_3_N_4_ and SiO_2_ layers were etched away by reactive ion etching process to expose the Si substrate, followed by KOH solution etching from the back side to fabricate a freestanding Si_3_N_4_ membrane which is transparent to electron beam in TEM. The etching time and temperature are kept at 1 hr and 80 °C, respectively. After etching, we cleaned the sample with D.I. water. A 25-nm-thick ZnO layer was then deposited on the top side of the chips by radio frequency magnetron sputtering at room temperature with pressure kept at 10 to 6 Torr and with an Ar/O ratio of 0.1. Subsequently, Au contact electrodes with a gap approaching 100 nm were fabricated by electron beam lithography with poly(methyl methacrylate) (PMMA) as photoresist, followed by metal deposition, and lift-off process. Finally, the TEM chip was loaded into the specially designed TEM holder, with which a bias can be directly applied through the Au electrodes inside the TEM.

### Measurements and characterizations

Crystal structures of the ZnO films was characterized with a Shimadzu X-ray diffractometer (XRD; Shimadzu, Kyoto, Japan), and grazing incident angle XRD with Cu Ka (*λ* = 0.154 nm) as the radiation source. *In situ* operation of the ReRAM devices, including forming, set, and reset processes, was investigated in an ultrahigh vacuum TEM (JEM-2000 V, JEOL Ltd., Tokyo, Japan) with a specially designed holder which is capable of applying electric current directly on the electrodes. Morphologies of the device and microstructures were investigated by field-emission scanning electron microscopy (FE-SEM; JSM-6500 F, JEOL) and transmission electron microscopy (JEM-3000 F, JEOL). The *I-V* characteristics of the MIM structure were measured with a Keithley 4200 semiconductor parameter analyzer (Keithley Instruments Inc., Cleveland, OH, USA) at room temperature in air.

## Results and discussion

**Figure 1:**
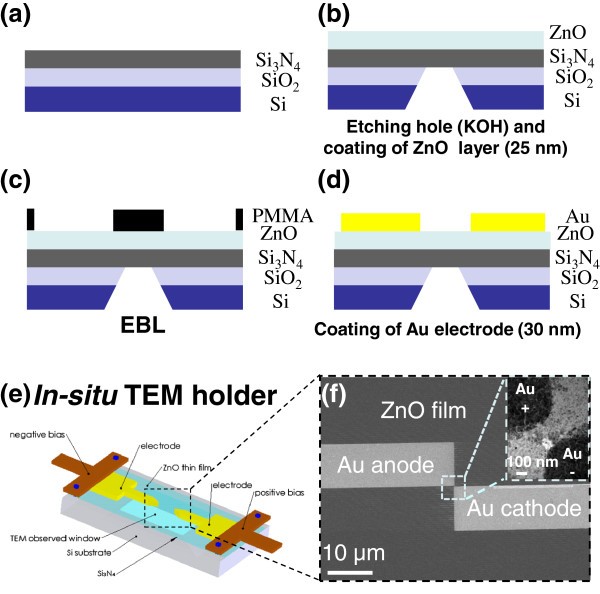
**Schematics of the TEM chip fabrication.** (**a**) Deposition of 80/20-nm Si_3_N_4_/SiO_2_ on Si substrate by LPCVD,(**b**) KOH chemical etching from the back side of the Si substrate, followed by deposition of 25-nm-thick ZnO layer by radio frequency magnetron sputtering, (**c**) E-beam lithography of PMMA to define position of the electrodes, (**d**) deposition of Au by metal deposition and lift-off processes, **e**) detailed configuration of the TEM chip after fabrication, and (**f**) the corresponding SEM and TEM images of the device.

Figure 1 shows the schematic fabrication processes of *in situ* TEM chip for the observation of the conducting bridge formation (See ESI in Additional file 1 for more details). Si_3_N_4_/SiO_2_ thin films with thicknesses of 80/20 nm were deposited on one side of a Si substrate by a LPCVD system. Position of the membrane area was defined by photolithography on the back side of chips. Si_3_N_4_ and SiO_2_ thin films were then etched away by reactive ion etching processes to expose the Si substrate for the subsequent KOH etching. A 25-nm-thick ZnO layer was then deposited on the top side of the chips by radio frequency magnetron sputtering at room temperature in order to obtain a smooth layer. X-ray spectrum with a sharp peak corresponding to (002) plane indicates that the ZnO film is of good crystallinity (ESI, Additional file 1: Figure S1). Au contact electrodes with a gap of approximately 190 nm were fabricated by electron beam lithography with PMMA as photoresist, followed by metal deposition and lift-off processes (Figure 1c). The overall configuration of the chip is shown in Figure 1d, for which the electron beam in the TEM can penetrate through the ZnO and Si_3_N_4_ layers to form images. The fabricated chip was loaded into the *in situ* TEM holder with a controllable bias applied inside the TEM, as shown in Figure 1e. The corresponding SEM and TEM images of the Au/ZnO/Au device are shown in Figure 1f and inset, respectively.

**Figure 2:**
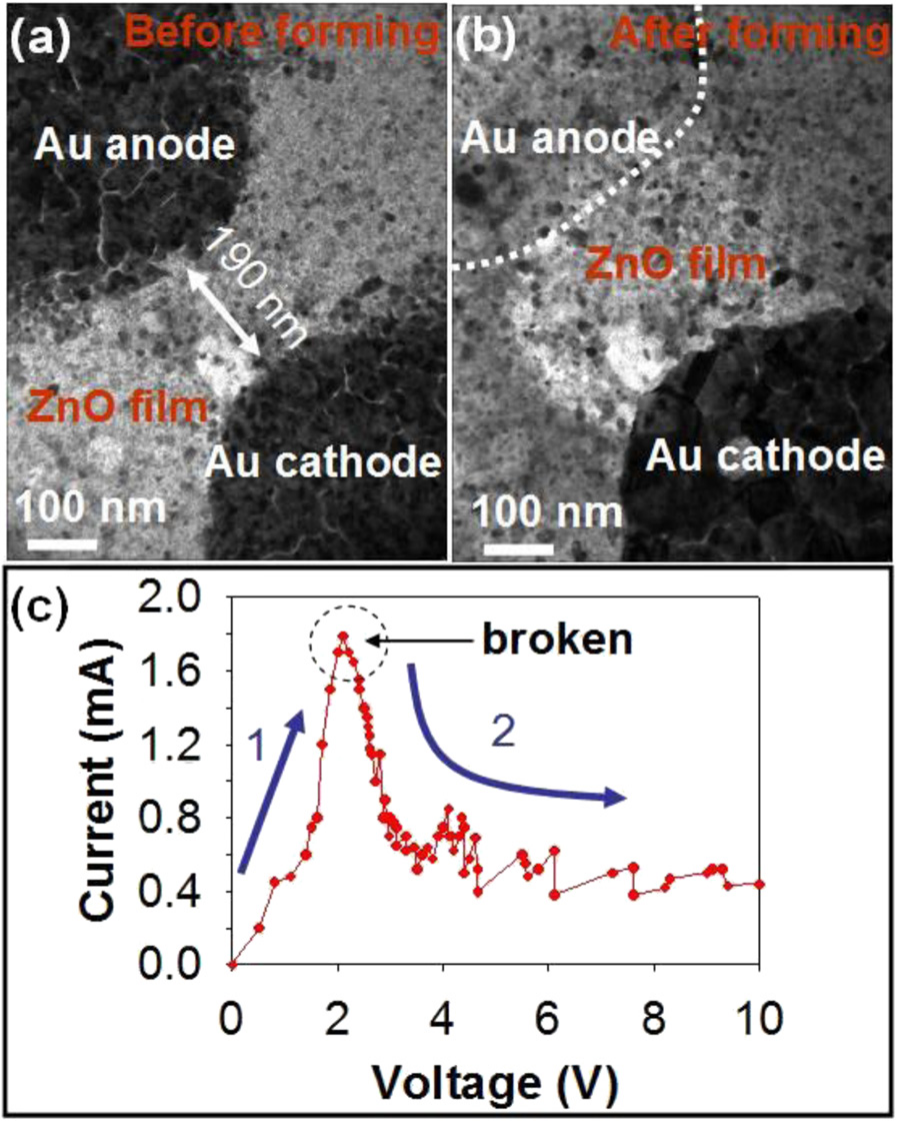
**TEM images of the Au/ZnO/Au device with a nanogap of 190 nm.**(**a**) Before and (**b**) after forming in a vacuum of <10^−9^ Torr. (**c**)The corresponding *I*-*V* behavior where the broken behavior could be seen.

For the ReRAM operation, the bias is applied to form the conducting bridge to turn the device from HRS to LRS, which is called a forming process. Figure 2a,b shows the TEM images of the Au/ZnO/Au device before and after the forming bias was applied in an ultrahigh vacuum <10^−9^ Torr. The corresponding *IV* behaviors are shown in Figure 2c. The current increases with the increasing of bias and reaches a maximum current of approximately 1.8 mA at a bias of approximately 2 V, while a sudden breakdown of the current occurs once bias is >2 V. The high conductivity of the ZnO film observed after the hard breakdown is ascribed to the opening of the conducting channel between the two Au electrodes due to the generation of defects, such as oxygen vacancies or Zn interstitials after the bias was applied, thereby a higher conductivity [18]. A high current due to high conductivity across two Au electrodes can readily lead to a significant migration of Au ions from the electrode, which agrees with the observation from the TEM image as shown in Figure 2b. Overall processes are irreversible owing to the lack of supplementary oxygen from the ambient to compensate the oxygen vacancies inside the ZnO layer in the reverse bias. 

**Figure 3:**
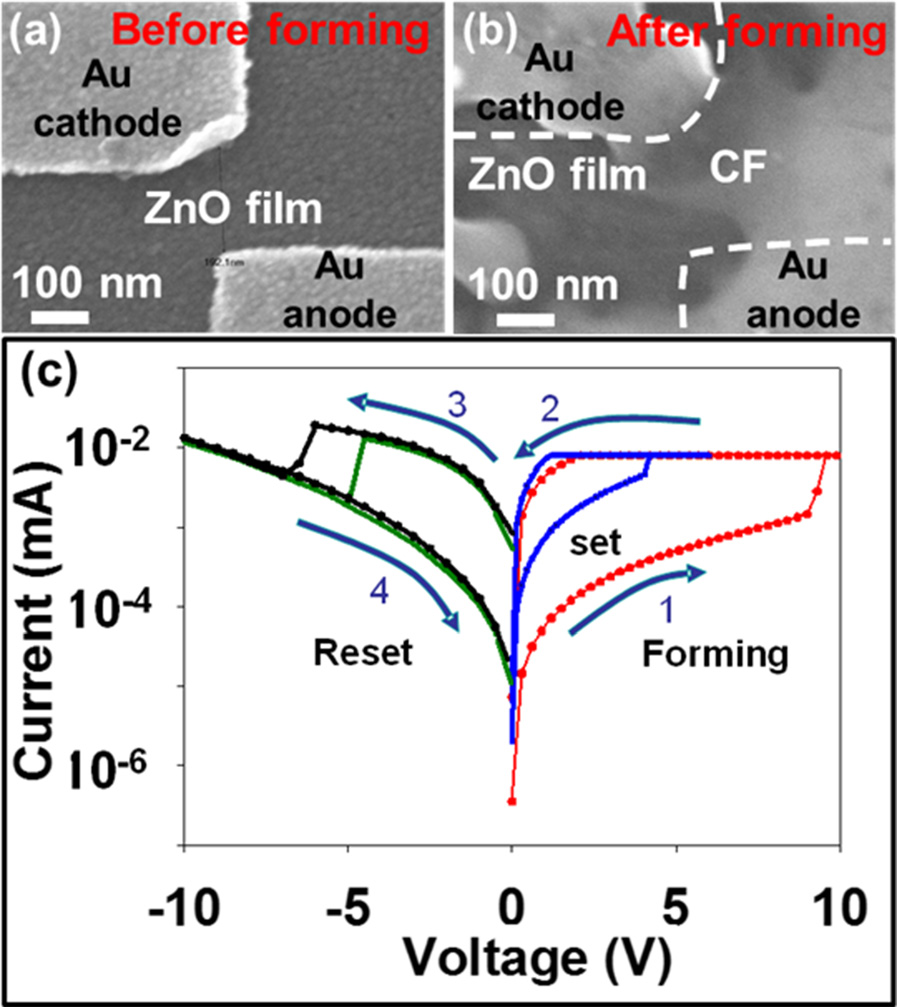
**SEM images of the An/ZnO/Au (a) before and (b) after forming in air.** (**c**) The corresponding *I*-*V* behavior shows the bipolar switching characteristic with forming, set, and reset voltages of approximately 8.5, 4.6, and 4.8 V, respectively.

Another device with the same fabrication processes was measured in air in order to prove the importance of environmental issue. The corresponding TEM images of the samples before and after the forming bias are shown in Figure 3a,b. Obviously, a typical bipolar switching *I*-*V* behavior could be observed after the forming process with forming, set, and reset voltages of approximately 8.5, 4.6, and 4.8 V, respectively, as shown in Figure 3c. Notably, the migration of Au ions from anode to cathode could be observed with a conical shape, indicating the formation of a conducting bridge (Figure 3b). The findings provide us the important information that the formation of a conducting bridge/path is more favorable in the ambient environment rather than in vacuum.

**Figure 4:**
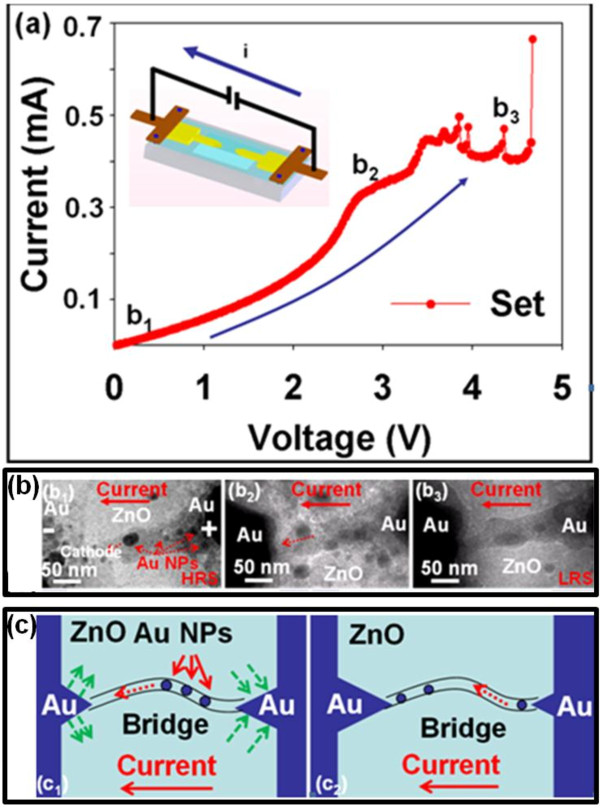
***I*-*V*curve of An/ZnO/Au operated at a set process (a).** The corresponding real-time images at b1, b2, and b3 in the *I*-*V* curve were recorded as (b1) to (b3) (**b**). (c1) and (c2) show the schematics of the conducting bridge formation associated with migration of Au NPs (**c**).

To understand the detailed formation of the conducting bridge between two electrodes, including the set and reset processes, the device after the forming process was loaded into the TEM for the *in situ* observation at different operation states. Therefore, a series of dynamic behaviors of the set and reset processes could be recorded simultaneously at different times with different biases. In order to ensure the accuracy of data recording, we have operated the device at set and reset processes several times. In the set process, the bias was applied into the device to switch the device from HRS to LRS, with which the sudden increase of current could be observed, as shown in Figure 4a. The inset shows the detailed configuration of the device and the direction of the applied current inside the *in situ* TEM. Figure 4b (b1 to b3) reveals the TEM images of dynamic switching behaviors from HRS to LRS at different applied biases, as indicated in the *IV* curve of Figure 4a. The corresponding schematics of the set processes are shown in Figure 4c (c1 and c2). Obviously, a substantial number of Au NPs in the ZnO layer can reduce the threshold voltages (set voltage) for the subsequent growth of the conducting bridge compared with that for the initial forming. We found that a conical path with different contrasts could be observed in the TEM image, which is expected from higher electron scattering behaviors due to a higher atomic number, containing Au NPs confirmed from the EDS spectrum as shown in Additional file 1: Figure S2 (ESI) by a local reduction-oxidation process from Au cations after an initial forming breakdown process. The results are similar to an electrochemical metallization (ECM) mechanism where the conducting bridge with the conical shape could be formed with a wide base from the cathode and a narrow neck near the anode [19]. Although, the formation of conducting bridge with a conical shape in the Au/ZnO/Au system agrees with the ECM mechanism, the formed positions of the conducting bridge for the wide base and the narrow neck are different from the typical formation of a conducting bridge via the ECM mechanism. In our case, the wide base of the conducting bridge was found near the anode, while the narrow neck was found near the cathode. Before the set process, a broken region near the cathode as shown in Figure 4b (image b1) could be clearly observed, which limits the current flow, providing us a distinct evidence of HRS. After the high-enough bias was applied (set process), Au cations could migrate from the interface of the anodic Au electrode to connect the broken region from the top region of the conducting bridge via the reduction/crystallization process, enabling the switching of resistive state (Figure 4b, b1 and c1). The higher the positive bias is applied, the more Au cations migrated to the cathode can be achieved (Figure 4b, b2). Finally, a complete bridge connected by two Au electrodes containing defective ZnO and Au NPs was observed at an applied bias of >4.6 V, namely a threshold voltage of LRS where an obvious increasing of current could be obtained (Figure 4b, b3 and Figure 4c, c2). In addition, the grain boundaries of the polycrystalline ZnO grains also promote the formation of the conducting bridge since Au ions preferentially accumulate at grain boundaries [20]. 

**Figure 5:**
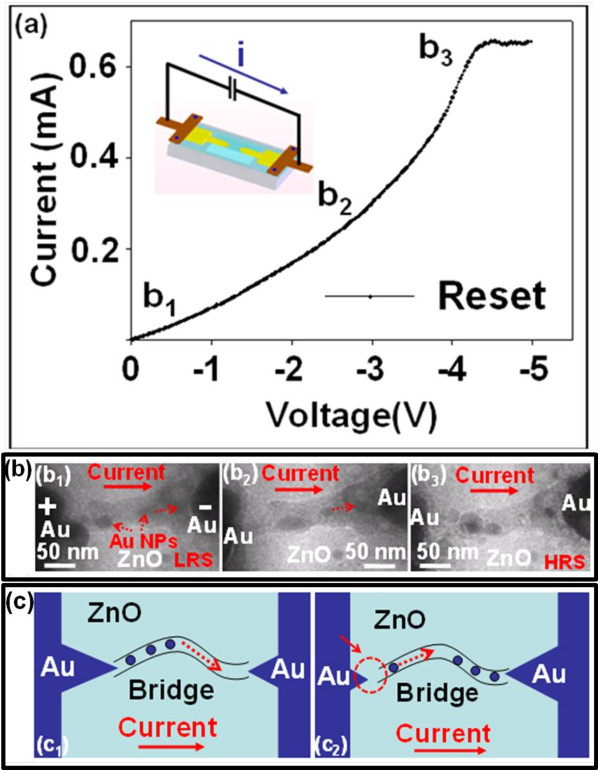
***I*-*V*curve of An/ZnO/Au operated at a reset process (a).** The corresponding real-time images at b1, b2, and b3 in the *I*-*V* curve were recorded as (b1) to (b3) (**b**). (c1) and (c2) show the schematics of the breakdown of the conducting bridge near the anode associated with the migration of Au NPs (**c**).

In the reset process, a reverse bias opposite the bias polarity of the set process is necessary to switch the device from LRS to HRS, as shown in Figure 5a. The corresponding device configuration with the direction of the current is shown in the inset of Figure 5a, for which an *in situ* dynamic reset process was recorded, as shown in Figure 5b (b1 to b3) at different biases as indicated by b1 to b3 in the corresponding *I*-*V* curve of Figure 5a. The schematics of the reset processes are shown in Figure 5c (c1 to c2). As the reverse bias increases, the current increases where the migration of Au cations from anode to cathode via the conducting bridge would occur again until a current saturation was observed at the reversed bias >4.5 V, indicating an occurrence of the reset processes (Figure 5b, b1 and b2, and Figure 5c, c1). The corresponding TEM image during the current saturation as shown in Figure 5b (b3) provides us with a distinct evidence that the migration of Au cations finally results in a discontinuity (broken region) near the interface of the anode and the narrow neck region, as marked by a dashed circle and arrow in Figure 5c, c2. The results are also consistent with our previous observation in the set process (Figure 4b, b1).

The formation of the conducting bridge from our *in situ* observation, which is opposite the conventional formation of the conducting bridge via the ECM mechanism, can be explained as due to the different diffusivity of cations transported inside the dielectric layer, thereby significantly influencing the morphology of the conducting bridge. For the conventional ECM mechanism, the matrix is typically composed of a solid electrolyte with a low melting point, for which cations, after an ionization process from the active electrode, can easily diffuse through the electrolyte to be reduced at the cathode with the wide base and then terminated at the anode with the narrow neck, resulting in the formation of the conducting bridge with the conical shape. In the typical ECM mechanism, the rate limit is determined by the reduction process at the interface of the cathode rather than the ion transport inside the electrolyte film. For the Au/ZnO/Au case as an example in our study, the rate limit is determined by the speed of diffusion for Au cations transported inside the ZnO film because the ZnO layer can be considered as a dense film, thereby limiting the diffusion of Au cations. Once Au cations ionized from Au electrode transport into the ZnO film, they will be reduced into Au NPs immediately, leading to the wide base of the conducting bridge near the anode and the formation of the narrow neck near the cathode, which is consistent with the results observed by other groups in Ag/SiO_2_/Pt [21] and Ag/ZrO_2_/Pt systems [22]. Furthermore, an issue regarding the formation of a conducting bridge on the surface of the ZnO or inside the ZnO film and the role of oxygen vacancies inside the ZnO for the formation and dissolution of the Au-conducting bridge have to be investigated systematically.

## Conclusions

In summary, we have designed a specific TEM chip for the *in situ* observation of ReRAM operations, including set and reset processes based on Au/ZnO/Au system inside a TEM. An obvious bipolar resistive switching behavior with a nanogap distance of 190 nm could be achieved in air with forming, set, and reset voltages of approximately 8.5, 4.6, and ~4.8 V, respectively. A clear conducting bridge associated with the migration of Au ions inside a defective ZnO film from cathode to anode via the conducting bridge during forward/reverse biases could be clearly observed by taking a series of consecutive TEM images, while the formed positions of the conducting bridge with a wide base and narrow neck is different from a typical formation of conducting bridge via the ECM mechanism. The difference can be ascribed to the different diffusivities of cations transported inside the dielectric layer, thereby significantly influencing the morphology of the conducting path. In addition, a broken region near the cathode after reset process was observed, while it will be reconnected after the set process, which provides a direct proof of the formation of the conducting bridge. The current TEM technique is quite unique and informative, which can be used to elucidate the dynamic processes in other devices in the future.

## Competing interests

The authors declare that they have no competing interests.

## Authors’ contributions

CNP deposited the gold electrode and ZnO film for the *in situ* TEM sample, e-beam writer, and measured the RRAM property. CWW operated the *in situ* TEM instrument. TCC designed the *in situ* TEM sample. WYC carried out the SEM characterization. HWT grew the *in situ* sample of Si_3_N_4_ film. YCW etched and observed the *in situ* sample. WWW supported the knowledge and database of the *in situ* TEM. LJC supported the *in situ* TEM instrument. YLC organized the final version of the paper. All authors read and approved the final manuscript.

## Supplementary Material

Additional file 1 **Electronic Supplementary Information (ESI).** Contains **Figure S1** and **Figure S2**. Click here for file
