# Supplementary material for: Resistive switching of Au/ZnO/Au resistive memory: an in situ observation of conductive bridge formation
Source: Nanoscale Res Lett. 2012 Oct 8;7(1):559. doi: 10.1186/1556-276X-7-559 (PMC3494527; doi:10.1186/1556-276X-7-559)
Supplement: Additional file 1 — Electronic Supplementary Information (ESI). Contains Figure S1 and Figure S2. [file 1556-276X-7-559-S1.doc]

**Resistive switching of Au/ZnO/Au resistive memory: An *in-situ* observation of conductive bridge formation***

**Chung-Nan Peng^a,c^, Tsung-Cheng Chan^a,c^, Wen-Yuan Chang^a,c^, Yi-Chung Wang^a,c^ Hung-Wei Tsai^a,c^, Chun-Wen Wang*^b^*, Wen-Wei Wu*^b^*, Lih-Juann Chen*^a^*, and Yu-Lun Chueh^*,a,c^**

^a^Department of Materials Science & Engineering, National Tsing Hua University, No. 101, Sec. 2, Kuang-Fu Rd., Hsinchu 30013, Taiwan, Republic of China.

^b^Department of Materials Science & Engineering, National Chiao-Tung University, No. 1001, University Rd., Hsinchu 30013, Taiwan, Republic of China.

^c^Center For Nanotechnology, Material Science, and Microsystem, National Tsing Hua University, No. 101, Sec. 2, Kuang-Fu Rd., Hsinchu 30013, Taiwan, Republic of China.

*E-mail: ylchueh@mx.nthu.edu.tw; Fax: +886-3-5722366; Tel: +886-3-5715131-33965*

**Figure S1 XRD spectrum of ZnO film.**


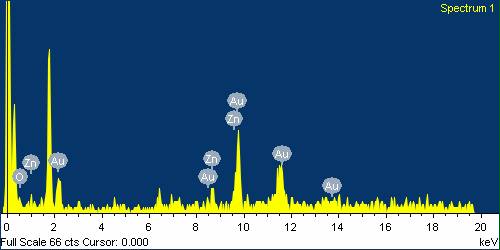


**Figure S2 Energy dispersive spectrum of Au NPs.**
